# Clinical Application of Antimicrobial Bone Graft Substitute in Osteomyelitis Treatment: A Systematic Review of Different Bone Graft Substitutes Available in Clinical Treatment of Osteomyelitis

**DOI:** 10.1155/2016/6984656

**Published:** 2016-01-21

**Authors:** T. A. G. van Vugt, J. Geurts, J. J. Arts

**Affiliations:** Department of Orthopaedic Surgery, Research School CAPHRI, Maastricht University Medical Centre, P.O. Box 5800, 6229 HX Maastricht, Netherlands

## Abstract

Osteomyelitis is a common occurrence in orthopaedic surgery, which is caused by different bacteria. Treatment of osteomyelitis patients aims to eradicate infection by debridement surgery and local and systemic antibiotic therapy. Local treatment increases success rates and can be performed with different antimicrobial bone graft substitutes. This review is performed to assess the level of evidence of synthetic bone graft substitutes in osteomyelitis treatment. According to the PRISMA statement for reporting systematic reviews, different types of clinical studies concerning treatment of osteomyelitis with bone graft substitutes are included. These studies are assessed on their methodological quality as level of evidence and bias and their clinical outcomes as eradication of infection. In the fifteen included studies, the levels of evidence were weak and in ten out of the fifteen studies there was a moderate to high risk of bias. However, first results of the eradication of infection in these studies showed promising results with their relatively high success rates and low complication rates. Due to the low levels of evidence and high risks of bias of the included studies, these results are inconclusive and no conclusions regarding the performed clinical studies of osteomyelitis treatment with antimicrobial bone graft substitutes can be drawn.

## 1. Introduction

### 1.1. Background

Osteomyelitis is an infection of bone or bone marrow with a concomitant inflammation involving the bone marrow and the surrounding tissues. These infections can originate from many different mechanisms [1]. In the many years that passed since the first reported osteomyelitis, the knowledge about these infections increased significantly [2]. Nowadays, we know that a common cause of osteomyelitis is a bacterial infection, for instance, the infection with* Staphylococcus aureus* after surgical intervention. Osteomyelitis is a major complication in orthopaedic surgery and is usually associated with open fracture surgery, bone reconstruction surgery, or orthopaedic implants [1]. Despite the numerous cases of osteomyelitis in orthopaedic surgery there is no precise epidemiological data available, but in literature the incidence of osteomyelitis ranges from 1% in primary hip replacement up to 55% in treatment of type III open fractures [3–6]. Appropriate treatment of patients with osteomyelitis is necessary because osteomyelitis can become chronic or is associated with delayed union or nonunion of fractures or failure of prosthesis implantation. In more severe untreated osteomyelitis bone sequestration, sinus formations or sepsis can cause disabling or life threatening complications [7].

In osteomyelitis treatment, eradication of the bacterial infection is essential. Acute osteomyelitis is often treated with systemic antibiotic administration, where chronic osteomyelitis treatment often requires surgical debridement in combination with local and systemic administration of broad-spectrum antibiotics like vancomycin, ciprofloxacin, and gentamicin [1]. Systemic administration of these antibiotics causes systemic side effects and the effects and concentrations at the infection site are low. Thereby, osteomyelitis compromises the local vascularity, which decreases the effects of oral or parenteral administration [8].

A higher delivery of antibiotics can be performed using local administration of antibiotics, which results in a major increase of the concentrations at the infection site. Some antibiotic carriers that can perform this local administration are antibiotic-loaded spacers, PMMA beads, or antibiotic-loaded bone graft substitutes like calcium sulphate pellets and collagen fleeces. Besides the antibiotic-loaded bone graft substitutes, there are synthetic bone graft substitutes without antibiotics but which do have antimicrobial capacities, like the bioactive glass S53P4 [9, 10].

The use of antibiotic-loaded or antimicrobial bone graft substitutes has advantages over nonresorbable antibiotic carriers, because of its biodegradability. This biodegradability enables a “one-stage” operative procedure, whereas use of the antibiotic-loaded spacers and PMMA beads/chains requires a “two-stage” operative procedure [11]. One-stage revision surgery is preferred over a two-stage revision surgery procedure, because of the lower burden to the patients, shorter hospitalization, lower infection risks, improved results of joint function, and lower costs [12]. Major drawbacks of one-stage revision surgery are the lower success rates and thereby it is not always possible to accurately plan the bone reconstruction part of the surgery [13, 14]. Where PMMA chains are deemed to be the golden standard in two-stage surgery, bone graft substitutes could become the golden standard in one-stage surgery and thereby they could be able to increase the success rates of this procedure [8].

There are numerous bone graft substitutes, which all differ in properties like biomaterial composition, mechanical properties, and biodegradability properties. These properties are important in processes involving bone proliferation and bone healing, like providing an ideal environment for osteoblast proliferation [15]. Most of these bone graft substitutes consist of calcium phosphates as tricalcium phosphate (TCP) or hydroxyapatite (HA), calcium sulphates (CS), or bioactive glass (BAG). The biodegradability, biocompatibility, and several other properties of these different materials have been described in clinical studies and these studies showed that using the bone graft substitutes in treatment of different bone defects gives good results [16].

Important properties in the usage for local antibiotic delivery are the antibiotic binding capacity, tissue penetration capacity, and the antibiotic release profile of the carrier. These properties are important because the antibiotic-loaded bone graft substitute has to reach local concentrations as high as possible without reaching environmental toxicity. Concentrations of probably 100 times the minimal inhibitory concentration (MIC) in the first 1-2 days are desirable, where the release concentration after several days may be significantly lower but remains bactericidal [17].

In conclusion, an ideal bone graft substitute should be biocompatible, biodegradable, and should provide osteoconduction and osteoinduction [16]. There are several commercial available products, which claim to be good antimicrobial bone graft substitutes for osteomyelitis treatment, namely, Osteoset-T^*®*^, Perossal^*®*^, BonAlive^*®*^, Herafill^*®*^ beads, Cerament-G^*®*^ and Stimulan^*®*^. However, a summary of clinical evidence or evidence-based guidelines for the application of these products, to assist surgeons in treatment of osteomyelitis, is not available.

### 1.2. Objectives

In this systematic review, according to PRISMA statement, the level of clinical evidence of different (antibiotic-loaded) synthetic bone graft substitutes for use in osteomyelitis treatment will be analysed, where the clinical outcome results will be assessed as well.

## 2. Methods

### 2.1. Protocol

In the development of this systematic review, the PRISMA statement for reporting systematic reviews and meta-analyses of studies that evaluate health care interventions has been followed [18].

### 2.2. Eligibility Criteria

#### 2.2.1. Types of Studies

All types of prospective and retrospective cohort and case-control studies concerning the clinical application of different commercially available (antibiotic-loaded) synthetic bone graft substitutes in treatment of osteomyelitis were assessed. Case reports or review papers were excluded. Due to the lack of clinical evidence, case reports were assessed. English, Dutch, and German language restriction was chosen.

#### 2.2.2. Patients and Interventions

Patients with any type of chronic osteomyelitis due to all causes were considered for inclusion. Furthermore, no restriction for gender and age was used.

Trials evaluating the usefulness of different types of (antibiotic-loaded) synthetic bone graft substitutes in the treatment of patients with osteomyelitis were included. This review is limited to studies using only commercially available antibiotic-loaded bone graft substitutes and studies using bioactive glass S53P4.

#### 2.2.3. Outcomes

Primary outcome measures focussed on the curative behaviour of the different bone graft substitutes. It was assessed if there was an eradication of the osteomyelitis. Secondary outcome measures are the biodegradability and the influence on bone regeneration of the bone graft substitute material.

### 2.3. Search Methods for Identification of Studies

For the identification of useful trials, several databases were used. Databases used for the selection of papers are the PubMed databases, the EMBASE database, and the Web of Science databases. Furthermore, reference list of the included articles was studied in the search for additional articles for inclusion.

The authors have searched in these different databases for papers of trials of AL (antibiotic-loaded) bone graft substitutes or bioactive glasses used in osteomyelitis treatment. In all databases, articles were searched using a systematic search including database specific keywords, brand or product names, and properties of the different products.

### 2.4. Data Collection and Analysis

Studies were selected based on the title and abstract by the assessment of two reviewers (TV and JA). The articles retrieved were judged by language where only English, Dutch, or German articles are included. Also articles were judged on the full text availability and the previously mentioned eligibility criteria.

The data obtained from the selected articles was scored and processed in different data collection forms or tables. One reviewer executed all data collection, where a second reviewer checked the extracted data. Information or variables sought were concerning study design, number and type of participants, patient characteristics, type of bone graft substitute, used antibiotic agent, and outcome measurements.

### 2.5. Bias

The level of evidence of the different articles was assessed by using the CEBM criteria for levels of evidence published by the Oxford Centre of Evidence and the methodological quality of the articles was assessed using the “Cochrane Collaboration's tool for assessing he risk of bias” [34, 35]. According to these criteria, the following types of bias were assessed: selection bias, performance bias, detection bias, reporting bias, and other potential sources of bias. Besides the assessment of bias, the authors also assessed the possibility of several confounding factors concerning participants and the intervention.

## 3. Results

### 3.1. Study Selection

**Figure 1:**
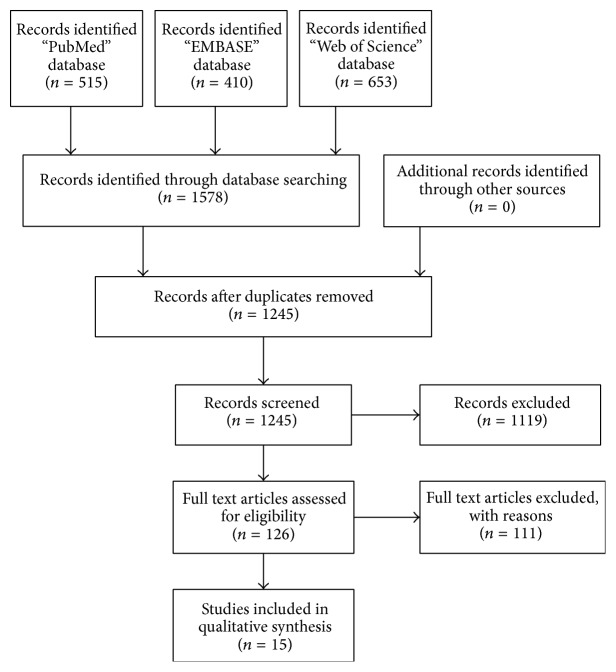
Study selection flow chart.

In this review, a total of 15 articles of 15 different studies were included for final analysis (Figure 1). The different search methods provided a total of 1578 records at first. Searching the PubMed database gave 515 results, whereas a comparable search in the EMBASE database resulted in 410 hits. The systematic search in the Web of Science databases resulted in 653 records. To be sure that no important trials or studies were missed, the complete search was also controlled and executed by a librarian of the Maastricht University. After adjusting all duplicates, this search gave 1245 results. These results were assessed on their titles and 1119 studies were discarded because of different reasons. Of the remaining 126 studies, 101 studies were excluded because the abstract showed they did not meet the eligibility criteria. After examining the full text articles of the 25 remaining articles, 10 articles were excluded because of different reasons. Three studies were excluded because they treated infected ulcers in diabetes treatment and no osteomyelitis; four studies were discarded because full text was not available; one study was excluded because the study only reported in vivo elution characteristics and the last two studies were excluded because they were congress abstracts without an article.

The total amount of 15 studies included seven studies where Osteoset-T was tested [19–23, 31, 32], four studies where BonAlive was tested [26–29], two studies where Perossal was tested [24, 25], and two studies where Herafill-G was tested [30, 33]. There was no clinical data found of the resulting Cerement-G and Stimulan. These products do have different chemical compositions and have different antimicrobial effects based on their working mechanism or different types of antibiotics; these characteristics are shown in Table 1.

### 3.2. Study Characteristics

Of all 15 studies selected for this review only McKee et al. performed a randomized controlled trial. Seven studies were performed as prospective nonrandomized clinical trials; four studies were performed as retrospective nonrandomized clinical trials and three studies were case reports. So randomization was only performed in one of the 15 studies and none of these studies were single- or double-blinded. Specific study details are available in the supplementary data in Supplementary Material available online at http://dx.doi.org/10.1155/2016/6984656.

The total amount of patients in the included studies was 484 (Table 2). These were patients suffering from acute or chronic osteomyelitis or patients with infected nonunion fractures. All patients were older than 16 years and the mean age ranges from 32 years to 59 years in the different studies, where the male/female ratio was 347/137.

Only three of all fifteen studies used a control group to compare their treatment [19, 22, 29]. In one study, Osteoset-T bone graft substitutes were compared with antibiotic PMMA beads and in another study osteomyelitis treatment with Osteoset-T combined with debridement was compared to only debridement treatment [19, 22]. In the third study, BonAlive bioactive glass was compared with two subgroups, one group with patient specific antibiotic-loaded Perossal pellets and one group with Teicoplanin loaded Calcibon granules mixed with Targobone [31]. In the remaining studies, the treatment of osteomyelitis with antibiotic-loaded bone graft substitutes or bioactive glass was not compared with any kind of control group.

All the 15 studies had comparable primary and secondary outcomes. In nine studies, eradication of infection was primary outcome and bone ingrowth/BGS degradation or complication rates were secondary outcomes. In three studies, bone growth/BGS degradation was the primary outcome and eradication of infection was the secondary outcome [24, 25]. One study had the pharmacokinetics of local administered gentamicin as primary outcome, where eradication of infection was a secondary outcome [30]. Outcome measurement was performed using radiological (X-ray or CT), haematological (erythrocyte sedimentation, CRP, or leucocyte concentration), serological (culture), or histological diagnostic techniques.

### 3.3. Risk of Bias of the Included Studies

The risk of bias within each study is evaluated using different guidelines from the Cochrane handbook for systematic reviews [34]. We customized the different guidelines stated for randomized and nonrandomized clinical trials to estimate all the different types of bias that are involved in different study designs. The risk factors of bias were divided into 5 different subjects, specifically patient selection and selection bias, quality of methodology, follow-up, reporting bias and confounders, and other sources of bias (potential related risks of bias due to study design or conflicts of interest). These risks of bias are scored as low (+ = 2), moderate (+/− = 1), or high (− = 0) (Table 3). Exact data and corresponding comments of the evaluation of risks of bias are available in the supplementary data.

In all studies included, patients had osteomyelitis or nonunion bone infection, but in most studies it was not mentioned how or why these patients were included. Most studies did not describe any specific inclusion or exclusion criteria that could cause selection bias. Thereby, only in three of the fifteen studies were control groups used, but only McKee et al. [19] used randomization of patients. In none of all the studies' patients were health care assessors or data collectors blinded.

Quality of the methodology was compared to see if there is not any performance bias or detection bias in the different studies. In almost all studies, outcome definitions were defined as eradication of infection, bone growth, and bone graft substitute degradation and in one study complication rates were a secondary outcome. Outcome measurement was commonly performed with radiological techniques (single radiograph or CT) and haematological test (erythrocyte sedimentation, leukocyte count, or CRP), which are all sufficient techniques to estimate the outcome. The studies differ in clearness of the protocol of treatment; in most studies, authors did not mention anything about the interventions and cointerventions performed.

Most studies tried to have a follow-up of patients of at least 24 months because in 24 months most cases of infection recurrence are seen. Many of these studies had a mean follow-up longer than the 24 months planned. In several studies, loss to follow-up did occur where most of the time the patients died, but in most cases this was not attributed to the intervention (Table 2).

The outcomes in most articles are clearly discussed but the outcome parameters or results of different test are not reported. Most of the authors did not use any parameters as CRP or erythrocyte sedimentation to define that infections were eradicated but they only mentioned that infection was eradicated in all or a part of the patients. The same problems of reporting data occurred at the bone growth and degradation of bone graft substitutes. Thereby, none of the studies described how they exactly measured bone growth and degradation of bone graft substitutes using imaging techniques.

In three studies, other probable risks of bias are seen, but in the other twelve studies no potential risk of other sources of bias is mentioned. In the first study, no conflict of interests was mentioned, but this study is used by the manufacturer as promotion material, which could increase the risk of different sources of bias. In the second study, the primary study seems to be for different operation techniques, but the results of treatment with bone graft substitutes are reported, where the third study aimed to investigate the local and systemic toxicity of gentamicin in Herafill-G beads, but had eradication of infection as a secondary outcome.

The 15 studies included in this review contain major differences from each other regarding multiple factors. Comparing the levels of evidence of all studies, the data shows that these levels vary from 1b to 4 (Table 4). Thereby, the risks of bias in the different studies vary from a score of 2/10 to 9/10 (Table 3). These differences are not solely seen when all studies are compared with each other, but when we differentiate per product the differences in level of evidence or risks of bias are not comparable. All the differences mentioned decrease the comparability of the included studies, which may affect the cumulative results and level of evidence negatively.

A striking example for the difficulty of comparing these studies is the lack of reporting outcome data and the absence of an intervention protocol in most studies. Most authors discussed their results and outcome, but the additional data and probable statistical analysis or significance are not mentioned in most cases.

### 3.4. Results of Individual Studies

Although the differences in the included studies were mentioned previously, the results of all different studies show some good results initially (Table 5). Because the reporting of outcome data and results was poor in almost all studies, it was difficult to assess the value and compare all outcomes. Thereby, the lack of statistical analysis and significance must be taken into consideration. Most authors only discussed their results without any substantiating data, which resulted in the absence of mean values, standard deviations, confidence intervals, or other important information about the primary and secondary outcomes.

The results on the primary outcome of this review, eradication of infection, are described as a percentage (or part) of patients where infection is absent after treatment in all studies. These outcomes range from 80% to 100% eradication in all patients, but only Chang et al. showed a significant outcome with the comparison of subgroups with the same osteomyelitis classification in their study. In this study, treatment of osteomyelitis with debridement and Osteoset-T was compared with only debridement treatment and showed an eradication of 94% versus 59% (*p* = 0.024) [22]. McKee et al. did not show a significant difference between the two groups of antibiotic impregnated PMMA beads versus Osteoset-T [19]. Romanò et al. compared BonAlive with two different custom-made antibiotic-loaded bone graft substitutes but there was no significant difference in eradication of infection between these three groups [29]. In all other studies, little to no statistical analysis was performed. Comparisons between the studies with a high or low amount of participants show that a higher group of participants has lower success rates, except for the study of Ferguson et al. where an eradication of 90.8% was shown in a study population of 195 cases treated with Osteoset-T (Table 5) [31].

Secondary outcome results are reported in the same way as the primary outcome results and in none of the included studies statistical significance of outcomes is proven or analysed (Table 5). Bone growth was reported as a rate of patients that had shown new bone formation and bone graft substitute ingrowth, but Chang et al. and Gitelis and Brebach described bone growth as a percentage of new formed bone in the bone defect that is filled with bone graft substitutes. The success rates of this secondary outcome are 100% in all studies except for one. Gitelis and Brebach have shown a success rate of 87.5% but this is because two patients died before bone growth could take place [20]. Another secondary outcome was degradation of the different bone graft substitutes, which was radiologically measured as a percentage of all beads degraded in the implanted area, in all studies. Some studies show full degradation of the bone graft substitutes [19–24], where other studies reported partial degradation [26–28] of pellets and the end of follow-up.

In multiple studies, complications were reported and the complication rate varied between 0% and 25%. Just like the primary and secondary outcomes, higher complication rates are reported in the bigger trials. Most of the complications were not directly connected to the usage of the bone graft substitutes but were surgical complications as thromboembolism or muscle flap complications. In several studies, extensive wound leakage is seen [26, 29, 31, 32]. Other reported complications were related with the patients' health status where von Stechow et al. reported treatment failure because of implant loosening due to osteoporosis and Drago et al. reported death due to pneumonia. Complications reported and related with the intervention were seen in the larger trails; where McKee et al. and Drago et al. showed infection recurrence or bone refracture.

It is apparent that these results show that there might not be any significant difference between the different products that are reviewed. The primary and secondary outcomes seem to be comparable for most studies. The only difference reported is the degradation time; the ceramic bone graft substitutes (Osteoset-T, Perossal, and Herafill beads G) are expected to degrade faster than bioactive glass (BonAlive); thereby, this difference in biomaterial degradation can be associated with a lower chance of prolonged wound leakage.

## 4. Discussion

### 4.1. Summary of Evidence

This systematic review of 15 studies was performed to assess the utility of anti-infective bone graft substitutes in osteomyelitis treatment. Overall, the evidence of these studies is not sufficiently robust to determine the effectiveness of these individual bone graft substitutes for antibiotic drug delivery.

The primary and secondary outcomes of the included studies show promising results with high clinical success rates at short-term follow-up. The rates of eradication of infection vary from 80% to 100%, the bone growth rates vary from 87.5% to 100%, and the bone graft substitute degradation is 100% in all studies except for one.

Despite these good results, the study designs, the number of patients included, and the level of evidence in most reported studies are weak. These weaknesses result in moderate to high risk of bias in ten of the 15 included studies. Interestingly, the studies with a higher amount of participants have lower success rates in eradication of infection and these studies are the also the studies with a lower risk of bias and higher level of evidence. The quality of methodology was poor in most studies and only one of the included studies showed a statistically significant outcome. Therefore, we concluded that the internal validity of the included studies is poor.

### 4.2. Limitations

The low number of included studies, the low levels of evidence, and the poor quality of most studies are the major limitations of this review. Due to the low number of included studies, the clinical evidence for treatment of osteomyelitis with anti-infective bone graft substitutes is poor and decision-making in evidence-based medicine seems to be impossible. Furthermore, the included number of patients in the studies was low and in only two of all studies were control groups used.

Besides the low number of studies included and the small numbers of patients included, most of the reviewed studies had poor methodological quality, resulting in relatively high risks of bias. Thereby, there was a lack of reported outcome data and statistical analysis in these studies, which made it difficult to make a clear statement of the cumulative results of the included studies.

One of the major issues emerging from the studies included in this review are the potential publication bias because smaller trials, like case reports and small clinical trials, showed some good results. This might suggest that the inclusion of these studies may have led to overestimation of the treatment effects.

## 5. Conclusions

The present review was performed to evaluate the clinical evidence of osteomyelitis treatment with anti-infective bone graft substitutes. The first results of the included studies imply that treatment of osteomyelitis with antibacterial bone graft substitutes could be a good option. The results on eradication of infection, bone growth, and degradation of bone graft substitute are worth mentioning with the high success rates. Although the included studies showed some good results, the generalizability of the results caution must be applied because of the low levels of evidence and the significant risks of bias. Taken together, these results are inconclusive and no conclusions regarding the clinical outcomes of osteomyelitis treatment with these bone graft substitutes can be drawn.

Further work needs to be undertaken to determine whether the results shown in the included studies are as promising as they seem. In particular, research of high methodological quality has to be performed. Randomized controlled trials with higher numbers of patients, control groups, and good statistical analysis could provide more definitive evidence for the usefulness of anti-infective bone graft substitutes in osteomyelitis treatment.

## Supplementary Material

In these supplementary materials you can find the additional information of this review. The used search terms for the databases are listed in appendix A. In appendix B you can find specific details of all included studies as study-type, inclusion criteria and exclusion criteria. Appendix C shows the extensive evaluation of bias of each single study including some explanatory notes. For other questions contact the correspondence address.

## Figures and Tables

**Table 1:** Characteristics of included products.

Product name	Composition	Antimicrobial mechanism	Antibiotic type
Osteoset-T	Alpha-hemihydrate calcium sulphate	Antibiotic impregnated bone graft substitute	Tobramycin
Perossal	Nanocrystalline hydroxyapatite (51.5%) and calcium sulphate (48.5%)	Antibiotic impregnated bone graft substitute	Different types of antibiotics (surgeon's choice)
BonAlive	Bioactive glass S53P4 (53% SiO_2_, 4% P_2_O_5_, 23% Na_2_O, and 20% CaO)	Release of surface ions causing increase of pH and osmotic pressure	None
Herafill-G	Calcium sulphate and calcium carbonate	Antibiotic impregnated bone graft substitute	Gentamicin

**Table 2:** Patient characteristics of the included studies.

Study	*N* total	*N* intervention (I)	*N* controls (C)	Age (years)	Gender m/f	Mean follow-up (moths)
McKee et al. 2010 [19]	30	15	15	44.1 (I) 45.6 (C)	11/4 (I) 10/5 (C)	38 (26–60)
Gitelis and Brebach 2002 [20]	6	6	0	50	3/3	28 (12–84)
von Stechow et al. 2005 [21]	16	16	0	? (38–85)	13/3	? (12–48)
Chang et al. 2007 [22]	65	25	40	39.8 (18–69)	36/29	75 (36–344)
Tsai et al. 2004 [23]	2	2	0	54 (80–28)	2/0	36
von Stechow and Rauschmann 2009 [24]	12	12	0	59 (39–74)	8/4	? (12–?)
Berner et al. 2008 [25]	1	1	0	49	1/0	24
Drago et al. 2013 [26]	27	27	0	44 (20–80)	18/9	17.8 (9–30)
Lindfors et al. 2010 [27]	11	11	0	? (16–84)	9/2	24 (10–38)
McAndrew et al. 2013 [28]	3	3	0	44.7 (26–68)	2/1	17.3 (14–21)
Romanò et al. 2014 [29]	76	27	49	45.2 (19–80)	49/27	21.8 (12–36)
Fleiter et al. 2014 [30]	20	20	0	51.1 (24–79)	16/4	?
Ferguson et al. 2014 [31]	193	193	0	46.1 (16–82)	150/43	44.4 (15.6–81.2)
Humm et al. 2014 [32]	21	21	0	49 (26–88)	18/3	16 (6–25)
Franceschini et al. 2012 [33]	1	1	0	32	1/0	12

**Table 3:** Overview of risks of bias of the included studies.

Study	Patient selection	Quality of methodology	Follow-up	Data reporting	Other	Total
McKee et al. 2010 [19]	2	2	2	1	2	**9**
Gitelis and Brebach 2002 [20]	1	2	1	0	2	**6**
von Stechow et al. 2005 [21]	0	1	2	2	1	**6**
Chang et al. 2007 [22]	1	2	2	1	2	**8**
Tsai et al. 2004 [23]	0	0	1	1	2	**2**
von Stechow and Rauschmann 2009 [24]	1	1	1	1	2	**6**
Berner et al. 2008 [25]	0	0	1	1	0	**2**
Drago et al. 2013 [26]	2	2	1	1	2	**8**
Lindfors et al. 2010 [27]	1	0	2	0	2	**5**
McAndrew et al. 2013 [28]	1	0	2	0	2	**5**
Romanò et al. 2014 [29]	1	2	2	2	1	**8**
Fleiter et al. 2014 [30]	1	0	0	2	0	**3**
Ferguson et al. 2014 [31]	1	1	2	2	2	**8**
Humm et al. 2014 [32]	0	1	2	0	0	**3**
Franceschini et al. 2012 [33]	0	1	1	0	1	**2**

**Table 4:** Levels of evidence and grades of recommendation.

Product name	Study	Level of evidence	Grade of recommendation
Osteoset-T	McKee et al. 2010 [19]	1b	B
	Gitelis and Brebach 2002 [20]	2b	
	Ferguson et al. 2014 [31]	2b	
	von Stechow et al. 2005 [21]	2b	
	Chang et al. 2007 [22]	2b	
	Humm et al. 2014 [32]	3b	
	Tsai et al. 2004 [23]	4	

Perossal	von Stechow and Rauschmann 2009 [24]	2b	C
	Berner et al. 2008 [25]	4	

BonAlive	Drago et al. 2013 [26]	2b	C
	Lindfors et al. 2010 [27]	2b	
	Romanò et al. 2014 [29]	2b	
	McAndrew et al. 2013 [28]	3b	

Herafill-G	Fleiter et al. 2014 [30]	3b	D
	Franceschini et al. 2012 [33]	4	

**Table 5:** Outcomes and complications of all included studies.

Study	Infection eradication in % of patients	Bone growth in % of patients	BGS degradation in % of patients	Complications
McKee et al. 2010 [19]	86%	100%	100%	5 patients with complications: 1x reinfection, 2x refracture, 1x wound infection, and 1x neuropraxia

Ferguson et al. 2014 [31]	90%	94%	100%	20 patients with complications: 9x fractures and 11x prolonged wound leakage

Gitelis and Brebach 2002 [20]	100%	100%	100%	No complication

von Stechow et al. 2005 [21]	87.50%	87.50%	100%	2 patients died due thromboembolism, 2x due to revision surgery (seroma), and 3x due to other complications

Humm et al. 2014 [32]	95.20%	Not mentioned	Not mentioned	7x prolonged wound leakage

Chang et al. 2007 [22]	80%	Not mentioned	100%	5x recurrent infection

Tsai et al. 2004 [23]	100%	100%	100%	No complications

von Stechow and Rauschmann 2009 [24]	100%	100%	100%	3x screw loosening due to osteoporosis and 1x venous thrombosis

Berner et al. 2008 [25]	100%	100%	100%	No complications

Drago et al. 2013 [26]	88.90%	100%	100%^[1]^	1 patient died due pneumonia, 2x due to infection recurrence, and 1x due to prolonged wound leakage

Romanò et al. 2014 [29]	92.60%	100%	100%	1x prolonged wound leakage, 1x fracture, and 1x venous thrombosis

Lindfors et al. 2010 [27]	100%	Not mentioned	Not mentioned	1x muscle flap complication and 1x remaining fracture

McAndrew et al. 2013 [28]	100%	Not mentioned	100%	No complications

Fleiter et al. 2014 [30]	80%	Not mentioned	100%	Not mentioned

Franceschini et al. 2012 [33]	100%	100%	100%	No complications

^[1]^In all patients, bioactive glass degradation or bone incorporation was seen, but BAG was still radiologically visible at 24 months.

## References

[1] Conterno L. O., Turchi M. D. (2013). Antibiotics for treating chronic osteomyelitis in adults. *The Cochrane Database of Systematic Reviews*.

[2] Lew P. D. P., Waldvogel P. F. A. (2004). Osteomyelitis. *The Lancet*.

[3] Hogan A., Heppert V. G., Suda A. J. (2013). Osteomyelitis. *Archives of Orthopaedic and Trauma Surgery*.

[4] Pollard T. C. B., Newman J. E., Barlow N. J., Price J. D., Willett K. M. (2006). Deep wound infection after proximal femoral fracture: consequences and costs. *The Journal of Hospital Infection*.

[5] Trampuz A., Widmer A. F. (2006). Infections associated with orthopedic implants. *Current Opinion in Infectious Diseases*.

[6] Widmer A. F. (2001). New developments in diagnosis and treatment of infection in orthopedic implants. *Clinical Infectious Diseases*.

[7] Parsons B., Strauss E. (2004). Surgical management of chronic osteomyelitis. *The American Journal of Surgery*.

[8] Geurts J., Chris Arts J. J., Walenkamp G. H. I. M. (2011). Bone graft substitutes in active or suspected infection. Contra-indicated or not?. *Injury*.

[9] Coraça-Huber D. C., Fille M., Hausdorfer J., Putzer D., Nogler M. (2014). Efficacy of antibacterial bioactive glass S53P4 against *S. aureus* biofilms grown on titanium discs in vitro. *Journal of Orthopaedic Research*.

[10] Kluin O. S., van der Mei H. C., Busscher H. J., Neut D. (2013). Biodegradable vs non-biodegradable antibiotic delivery devices in the treatment of osteomyelitis. *Expert Opinion on Drug Delivery*.

[11] Kanellakopoulou K., Giamarellos-Bourboulis E. J. (2000). Carrier systems for the local delivery of antibiotics in bone infections. *Drugs*.

[12] El-Husseiny M., Patel S., MacFarlane R. J., Haddad F. S. (2011). Biodegradable antibiotic delivery systems. *Journal of Bone and Joint Surgery—Series B*.

[13] Leonard H. A. C., Liddle A. D., Burke Ó., Murray D. W., Pandit H. (2014). Single- or two-stage revision for infected total hip arthroplasty? A systematic review of the literature. *Clinical Orthopaedics and Related Research*.

[14] Wolf C. F., Gu N. Y., Doctor J. N., Manner P. A., Leopold S. S. (2011). Comparison of one and two-stage revision of total hip arthroplasty complicated by infection: a Markov expected-utility decision analysis. *The Journal of Bone and Joint Surgery—American Volume*.

[15] Saravanapavan P., Jones J. R., Verrier S. (2004). Binary CaO-SiO_2_ gel-glasses for biomedical applications. *Bio-Medical Materials and Engineering*.

[16] Van Der Stok J., Van Lieshout E. M. M., El-Massoudi Y., Van Kralingen G. H., Patka P. (2011). Bone substitutes in the Netherlands—a systematic literature review. *Acta Biomaterialia*.

[17] Walenkamp G. H. I. M., Vree T. B., van Rens T. J. G. (1986). Gentamicin-PMMA beads. Pharmacokinetic and nephrotoxicological study. *Clinical Orthopaedics and Related Research*.

[18] Liberati A., Altman D. G., Tetzlaff J. (2009). The PRISMA statement for reporting systematic reviews and meta-analyses of studies that evaluate health care interventions: explanation and elaboration. *Italian Journal of Public Health*.

[19] McKee M. D., Li-Bland E. A., Wild L. M., Schemitsch E. H. (2010). A prospective, randomized clinical trial comparing an antibiotic-impregnated bioabsorbable bone substitute with standard antibiotic-impregnated cement beads in the treatment of chronic osteomyelitis and infected nonunion. *Journal of Orthopaedic Trauma*.

[20] Gitelis S., Brebach G. T. (2002). The treatment of chronic osteomyelitis with a biodegradable antibiotic-impregnated implant. *Journal of Orthopaedic Surgery*.

[21] von Stechow D., Scale D., Rauschmann M. A. (2005). Minimizing the surgical approach in patients with spondylitis. *Clinical Orthopaedics and Related Research*.

[22] Chang W., Colangeli M., Colangeli S., Di Bella C., Gozzi E., Donati D. (2007). Adult osteomyelitis: debridement versus debridement plus Osteoset T pellets. *Acta Orthopaedica Belgica*.

[23] Tsai Y.-H., Huang T.-J., Shih H.-N., Hsu R. W.-W. (2004). Treatment of infected tibial nonunion with tobramycin-impregnated calcium sulfate: report of two cases. *Chang Gung Medical Journal*.

[24] von Stechow D., Rauschmann M. A. (2009). Effectiveness of combination use of antibiotic-loaded PerOssal with spinal surgery in patients with spondylodiscitis. *European Surgical Research*.

[25] Berner A., Linde H. J., Schubert T., Nerlich M., Englert C. (2008). Treatment of lower limb osteomyelitis by a local bone substitute supplemented with antibiotics. *Zeitschrift fur Orthopadie und Unfallchirurgie*.

[26] Drago L., Romanò D., De Vecchi E. (2013). Bioactive glass bag-S53P4 for the adjunctive treatment of chronic osteomyelitis of the long bones: an in vitro and prospective clinical study. *BMC Infectious Diseases*.

[27] Lindfors N. C., Hyvönen P., Nyyssönen M. (2010). Bioactive glass S53P4 as bone graft substitute in treatment of osteomyelitis. *Bone*.

[28] McAndrew J., Efrimescu C., Sheehan E., Niall D. (2013). Through the looking glass; Bioactive glass S53P4 (BonAlive^®^) in the treatment of chronic osteomyelitis. *Irish Journal of Medical Science*.

[29] Romanò C. L., Logoluso N., Meani E. (2014). A comparative study of the use of bioactive glass S53P4 and antibiotic-loaded calcium-based bone substitutes in the treatment of chronic osteomyelitis: a retrospective comparative study. *The Bone & Joint Journal*.

[30] Fleiter N., Walter G., Bosebeck H. (2014). Clinical use and safety of a novel gentamicin-releasing resorbable bone graft substitute in the treatment of osteomyelitis/osteitis. *Bone & Joint Research*.

[31] Ferguson J. Y., Dudareva M., Riley N. D., Stubbs D., Atkins B. L., McNally M. A. (2014). The use of a biodegradable antibiotic-loaded calcium sulphate carrier containing tobramycin for the treatment of chronic osteomyelitis: a series of 195 cases. *The Bone & Joint Journal*.

[32] Humm G., Noor S., Bridgeman P., David M., Bose D. (2014). Adjuvant treatment of chronic osteomyelitis of the tibia following exogenous trauma using OSTEOSET^®^-T: a review of 21 patients in a regional trauma centre. *Strategies in Trauma and Limb Reconstruction*.

[33] Franceschini M., Di Matteo A., Bösebeck H., Büchner H., Vogt S. (2012). Treatment of a chronic recurrent fistulized tibial osteomyelitis: administration of a novel antibiotic-loaded bone substitute combined with a pedicular muscle flap sealing. *European Journal of Orthopaedic Surgery and Traumatology*.

[34] Higgins J. P. T., Green S., The Cochrane Collaboration (2008). *Cochrane Handbook for Systematic Reviews of Interventions*.

[35] Heneghan C. (2009). EBM resources on the new CEBM website. *Evidence-Based Medicine*.

