# Supplementary material for: Clinical Application of Antimicrobial Bone Graft Substitute in Osteomyelitis Treatment: A Systematic Review of Different Bone Graft Substitutes Available in Clinical Treatment of Osteomyelitis
Source: Biomed Res Int. 2016 Jan 21;2016:6984656. doi: 10.1155/2016/6984656 (PMC4745864; doi:10.1155/2016/6984656)
Supplement: Supplementary file 1 — In these supplementary materials you can find the additional information of this review. The used search terms for the databases are listed in appendix A. In appendix B you can find specific details of all included studies as study-type, inclusion criteria and exclusion criteria. Appendix C shows the extensive evaluation of bias of each single study including some explanatory notes. For other questions contact the correspondence address. [file 6984656.f1.pdf]

Appendices

Appendix A: Search keywords

	Database specific keywords	Other keywords
**Disease**	Osteomyelitis, Bacterial infection, osteitis	Chronic osteomyelitis, Bone infection,
**Intervention & materials**	Drug delivery systems, drug carriers, anti-bacterial agent, Bone substitutes, Local anti-infective agents, Calcium sulphate, Calcium phosphate, calcium carbonate	Antibiotic-loaded bone substitute, bioactive glass, S53P4, local antibiotic therapy, Perossal, Bonalive, Herafill, Cerament, Osteoset, Stimulan
**Antibiotics**	Tobramycin, Vancomycin, Gentamycin, Clindamycin	Tobramycin, Vancomycin, Gentamycin, Clindamycin
**Study design**	Randomized controlled trial, clinical trials	Randomized clinical trials, randomized controlled trial, clinical trial

Table 1: Search terms systematic PubMed search

Appendix B: Included studies

Product name	Study	Study Design	Inclusion criteria	Exclusion Criteria
**Osteoset-T ®**	McKee 2010	Randomized controlled trial	Osteomyelitis patients or infected non-union patients, >16 years, symptoms present >90 days, presence necrotic bone & cultured bacteria.	Immunocompromised, pregnant, mentally incompetent, uncontrolled diabetes, aminoglycoside hypersensitivity patients, patients with degenerative bone, severe vascular or neurologic disease, Myasthenia Gravis, neurotoxic drugs
	Gitelis 2002	Prospective non-randomized clinical trial	Patients with radiological evidence of osteomyelitis.	-
	Ferguson 2014	Prospective non-randomized clinical trial	Patients with clinical, radiological and by culture confirmed chronic osteomyelitis	-
	von Stechow 2005	Retrospective non-randomized clinical trial	Patients with spondylitis in thoracic or lumbar spine	-
	Chang 2007	Retrospective non-randomized clinical trial	Patients with chronic osteomyelitis	-
	Humm 2014	Retrospective non-randomized clinical trial	Patients witch chronic osteomyelitis	-
	Tsai 2004	Case-report	Patients with infected tibial non-union fractures	-
**PerOssal ®**	Von Stechow 2009	Prospective non-randomized clinical trial	Patients with acute or chronic spondylodiscitis	-
	Berner 2008	Case-report	Patient with osteomyelitis	-
**Bonalive ®**	Drago 2013	Prospective non-randomized clinical trial	Presence of osteomyelitis of a long bone for at last 6 months, patient required debridement and bone defect filling, age >18 years,	Need for local plastic procedures, segmental bone defects, associated septic arthritis
	Lindfors 2010	Prospective non-randomized clinical trial	Patients with radiological diagnosed osteomyelitis	-

**Herafill-G ®**	Romano 2014	Retrospective non-randomized clinical trial	Patients with clinical, laboratorial and radiological confirmed chronic osteomyelitis	Segmental bone defects, associated septic arthritis need for concomitant plastic surgery.
	Mc Andrew	Prospective non-randomized clinical trial	Patients with radiological and histological diagnosed osteomyelitis	-
	Fleiter 2014	Prospective non-randomized clinical trial	Patients with osteitis or osteomyelitis >18 years	People with gentamicin related risk factors (renal impairment, allergy etc.)
	Franceschini 2012	Case-report	One patient with chronic tibial osteomyelitis	-

Table 2: Included studies per produc

Appendix C: Risks of bias per study

McKee 2010	Judgement	Support
**Patient selection & selection bias**	+	30 patients with osteomyelitis or bone infection of one hospital were included. Thereby inclusion and exclusion criteria and methods of randomization are clear. Control group and intervention group are comparable. No blinding performed.
**Quality of methodology**	+	Clear definition of primary and secondary outcomes and reliable outcome measurement. In the execution of this study a clear protocol is used.
**Follow-up**	+	Good follow-up period of 24 months. Loss to follow-up of one person in each group, not related to treatment or disease. No study contamination or bad therapeutic compliance reported.
**Data reporting & confounding**	+/-	All outcomes are clearly discussed but the exact data and outcome parameters are missing. Authors tried to reduce the confounding by participants or interventions. No statistical analysis.
**Other**	+	Low risks of other sources of bias.

Table 3: Risk of bias McKee et al. 2010

Gitelis 2009	Judgement	Support
**Patient selection & selection bias**	+/-	6 Patients with osteomyelitis of one hospital are included, but other inclusion or exclusion criteria are not reported. There is no control group and no randomization. Patient group includes participant with large variety in health and disease status.
**Quality of methodology**	+	Clear outcome definition and good techniques for outcome measurement.
**Follow-up**	+/-	No loss to follow up reported. One patient received an additional antibiotic because of MRSA infection.
**Data reporting & confounding**	-	Results are clearly discussed but general outcome parameters are not reported and CI's are missing. No confounding factors in participants or intervention. No statistical analysis performed.
**Other**	+	Low risk of other sources of bias

Table 4: Risk of bias Gitelis et al. 2009

Ferguson 2014	Judgement	Support
**Patient selection & selection bias**	+/-	195 cases of osteomyelitis in one hospital included, good inclusion criteria and participant selection. No clear exclusion criteria and no randomization or control groups.
**Quality of methodology**	+/-	Outcome definitions are clearly defined, but outcome measurement needed more specific definition. There was a clear study protocol in the execution of this study.
**Follow-up**	+	Good definitions for follow-up. Loss-to-follow-up clearly explained. Good therapeutically compliance and no specific suspicion for contamination.
**Data reporting & confounding**	+	Detailed outcome reporting with statistical analysis (no significant difference), limited explanation of probable confounders.
**Other**	+	Low risks of other sources of bias.

Table 8: Risk of bias Ferguson et al. 2014

von Stechow 2005	Judgement	Support
**Patient selection & selection bias**	-	Retrospective study. 32 patients with spondylitis included, but only 16 patients with a dorsal chirurgical treatment reported, reason unclear. No control group and no randomization.
**Quality of methodology**	+/-	Primary outcomes not clearly defined. Secondary outcomes are clear. Good measurement techniques. Clear treatment protocol.
**Follow-up**	+	2 patients died after treatment due to embolism. To other patients received additional antibiotics because of MRSA and TBC. 12 patients had good follow op.
**Data reporting & confounding**	+	Results are discussed but outcome parameters are missing. Good reporting of differences in health status and influences on results. No confounding and no statistical analysis.
**Other**	+/-	This study seems to be primary focussed on different surgical techniques but this article is based on performance with bone-graft substitutes.

Table 9: Risk of bias van Stechow et al. 2005

Chang 2007	Judgement	Support
**Patient selection & selection bias**	+/-	65 patients included with osteomyelitis diagnosed by the Cierny-Mader classification. The control group (40 patients) was not comparable with treatment group (25 patients). No randomization.
**Quality of methodology**	+	Good definition of primary and secondary outcomes. Good measurement techniques. Report of protocol used in study used subgroups to compare groups
**Follow-up**	+	No loss to follow up. No contamination. Good therapeutic compliance.
**Data reporting & confounding**	+/-	Outcomes discussed. Specific parameters not reported. Good in between group comparison. Statistical analyses performed but not discussed.
**Other**	+	Subgroups are analysed, which did show some significant positive results in patients treated with bone graft substitute when groups were comparable.

Table 10: Risk of bias Chang et al 2007

Humm 2014	Judgement	Support
**Patient selection & selection bias**	-	21 patients included without clearly defined inclusion or exclusion criteria. Thereby patient selection isn't completely mentioned. There is no randomization and there are no control groups.
**Quality of methodology**	+/-	Clearly defined outcomes and logical outcome measurement techniques. Good reasonable treatment protocol, but could defined more specific. There was no blinding.
**Follow-up**	+	Follow-up and loss to follow-up is clearly defined and analyzed. Therapeutic compliance and contamination not mentioned explicitly.
**Data reporting & confounding**	-	Outcome data reporting is poor. There is little to none statistical analysis.
**Other**	-	BGS is only tested at a specific infected location (only tibia), which can effect the outcomes.

Table 11: Risk of bias Humm et al. 2014

Tsai 2004	Judgement	Support
**Patient selection & selection bias**	-	Case report of two patients with osteomyelitis or non-union bone infection. No inclusion or exclusion criteria mentioned. No control group or randomization.
**Quality of methodology**	-	In this study not patients are treated with antibiotic PMMA beads previously to the Osteoset-T administration.
**Follow-up**	+/-	No previous follow-up period defined. Patients showed good results in follow up. No loss to follow up.
**Data reporting & confounding**	+/-	Outcomes are clearly discussed and outcome parameters are reported.
**Other**	-	It is a case report.

Table 12: Risk of bias Tsai et al. 2004

von Stechow 2009	Judgement	Support
**Patient selection & selection bias**	+/-	19 patients included with acute or chronic osteomyelitis. No inclusion or exclusion criteria defined Patients had a comparable health status at intervention start.
**Quality of methodology**	+/-	Good definition of primary and secondary outcomes. Outcome measurement mentioned but unclear.
**Follow-up**	+/-	Only reported the 12 patients with at least 12 months follow-up instead of the 19 included patients. No loss to follow-up.
**Data reporting & confounding**	+/-	Results poorly reported, no outcome parameters in article. Probably confounding factors of patients or interventions are avoided. No statistical analysis performed.
**Other**	+	Low risk of other sources of bias.

Table 13: Risk of bias von Stechow et al. 2009

Berner 2008	Judgement	Support
**Patient selection & selection bias**	-	Case report of a single patient with osteomyelitis. No inclusion or exclusion criteria mentioned. No control group or randomization.
**Quality of methodology**		No clear inclusion or exclusion criteria described.
**Follow-up**	+/-	Goof follow-up. No complications.
**Data reporting & confounding**	+/-	Pre-operative patients characteristics are well reported, but results after intervention are limited. No statistical analysis performed.
**Other**	-	It is a case report.

Table 14: Risk of bias Berner et al. 2008

Drago 2013	Judgement	Support
**Patient selection & selection bias**	+	27 patients with at least 6 months of chronic osteomyelitis are included. Clear inclusion and exclusion criteria and patients are comparable.
**Quality of methodology**	+	Good and clear outcome definitions and adequate measurement. Good follow-up period of 24 months defined.
**Follow-up**	+/-	Loss to follow-up of 1 patient. Patient died due to pneumonia.
**Data reporting & confounding**	+/-	Pre-operative patients characteristics are well reported, but results after intervention are limited. No statistical analysis performed.

**Other**	+	Good in vitro approval of used bone graft substitutes and no risks of other sources of bias.

Table 15: Risk of bias Drago et al. 2013

Lindfors 2010	Judgement	Support
**Patient selection & selection bias**	+/-	11 patients included with radiological diagnosed osteomyelitis where previous treatment did fail. Multicentre study. No further inclusion or exclusion criteria defined.
**Quality of methodology**	-	Outcome and outcome measurement are antibacterial properties according to clinical symptoms defined by a orthopaedic surgeon. No previous follow-up period defined.
**Follow-up**	+	No loss to follow-up. Mean follow up period was 24 months.
**Data reporting & confounding**	-	Outcomes are discussed but clear data was not described. Thereby authors did not describe any confounding factors. No statistical analysis performed.
**Other**	+	No risks of other sources of bias.

Table 16: Risk of bias Lindfors et al. 2010

Romano 2014	Judgement	Support
**Patient selection & selection bias**	+/-	76 patients included in a retrospective controlled trial with three different groups (2 control groups with different BGS). Clearly defined inclusion and exclusion criteria and well described patient selection. No randomization.
**Quality of methodology**	+	Recurrent infection and side effects are clearly defined outcomes and measurement techniques are clear. There is a detailed treatment protocol, but there is no blinding.
**Follow-up**	+	Clear follow-up protocol, good analyzed loss to follow-up. Treatment compliance and contamination did not appear.
**Data reporting & confounding**	+	Pre- and postoperative data reporting is good and detailed. Data analysis did not showed major significant differences between subgroups, but was performed correct.
**Other**	+/-	Exclusion of larger defect or complicated bone defect could influence the outcome reliability.

Table 17: Risk of bias Romano et al. 2014

McAndrew 2012	Judgement	Support
**Patient selection & selection bias**	+/-	3 patients with radiological and histological diagnosed osteomyelitis are included. Further inclusion or exclusion criteria are not defined. Different disease status between patients.
**Quality of methodology**	-	Outcomes and outcome measurement methods are not clearly defined.
**Follow-up**	+	No loss to follow-up. Mean follow-up period was 17,4 months.
**Data reporting & confounding**	-	Results are discussed, but no data or outcome parameters are reported. Thereby nothing is said about confounding factors. No statistical analysis performed.
**Other**	+/-	No risks of other sources of bias.

Table 18: Risk of bias McAndrew et al. 2012

Fleiter 2014	Judgement	Support
**Patient selection & selection bias**	+/-	Open label phase 2 pharmaceutical trial with 20 patients. Inclusion and exclusion criteria ar well formulated unlike participant selection methods. No randomization and no control groups.
**Quality of methodology**	+/-	Outcomes are clearly defined and outcome measurement is well substantiated. There is a good treatment protocol, but there is no blinding.
**Follow-up**	-	Follow-up is short and specified for toxicity of gentamicin. There is a loss to follow-up worth mentioning and this is not clearly substantiated or analyzed. Thereby there could be contamination.
**Data reporting & confounding**	+/-	Good outcome reporting and statistical analysis, but no analysis of the eradication outcomes. Potential confounders are not mentioned.
**Other**	-	Primary aims of this study are related with toxicity of Gentamicin and not with eradication of infection.It is a phase 2 study

Table 19: Risk of bias Fleiter et al. 2014

Franseschini 2012	Judgement	Support
**Patient selection & selection bias**	-	Case report of 1 patient with osteomyelitis. No inclusion or exclusion criteria.
**Quality of methodology**	+/-	Good defined primary and secondary outcomes. Adequate measurement of outcomes.
**Follow-up**	+/-	Short follow up of 12 months. No confounding factors concerning patient or treatment reported or discussed.
**Data reporting & confounding**	-	Only outcome is discussed, but there are no outcome parameters or results reported.
**Other**	-	It is a case report.This article is use as promotion material by manufacturer.

Table 20 Risk of bias Franceschini et al. 2012

Study	Patient selection	Quality of Methodology	Follow-up	Data reporting	Other	Total
McKee 2010	2	2	2	1	2	**9**
Gitelis 2002	1	2	1	0	2	**6**
Von Stechow 2005	0	1	2	2	1	**6**
Chang 2007	1	2	2	1	2	**8**
Tsai 2004	0	0	1	1	2	**2**
Von Stechow 2009	1	1	1	1	2	**6**
Berner 2008	0	0	1	1	0	**2**
Drago 2013	2	2	1	1	2	**8**
Lindfors 2010	1	0	2		2	**5**
McAndrew 2010	1	0	2	0	2	**5**
Romano 2014	1	2	2	2	1	**8**
Fleiter 2014	1	0	0	2	0	**3**
Furgeson 2014	1	1	2	2	2	**8**
Humm 2013	0	1	2	0	0	**3**
Franceschini 2012	0	1	1	0	1	**2**

Table 21: Cumulative results risks of bias included studies
